# Radiation-Induced Brain Injury: Mechanistic Insights and the Promise of Gut–Brain Axis Therapies

**DOI:** 10.3390/brainsci14121295

**Published:** 2024-12-23

**Authors:** Mengting Li, Fan Tong, Bian Wu, Xiaorong Dong

**Affiliations:** 1 Cancer Center, Union Hospital, Tongji Medical College, Huazhong University of Science and Technology, Wuhan 430022, China; 2 Hubei Key Laboratory of Precision Radiation Oncology, Wuhan 430022, China; 3 Institute of Radiation Oncology, Union Hospital, Tongji Medical College, Huazhong University of Science and Technology, Wuhan 430022, China

**Keywords:** radiation-induced brain injury, radiotherapy, gut–brain axis, gut microbiota, therapeutic strategies

## Abstract

Radiation therapy is widely recognized as an efficacious modality for treating neoplasms located within the craniofacial region. Nevertheless, this approach is not devoid of risks, predominantly concerning potential harm to the neural structures. Adverse effects may encompass focal cerebral necrosis, cognitive function compromise, cerebrovascular pathology, spinal cord injury, and detriment to the neural fibers constituting the brachial plexus. With increasing survival rates among oncology patients, evaluating post-treatment quality of life has become crucial in assessing the benefits of radiation therapy. Consequently, it is imperative to investigate therapeutic strategies to mitigate cerebral complications from radiation exposure. Current management of radiation-induced cerebral damage involves corticosteroids and bevacizumab, with preclinical research on antioxidants and thalidomide. Despite these efforts, an optimal treatment remains elusive. Recent studies suggest the gut microbiota’s involvement in neurologic pathologies. This review aims to discuss the causes and existing treatments for radiation-induced cerebral injury and explore gut microbiota modulation as a potential therapeutic strategy.

## 1. Introduction

Radiation therapy is pivotal in managing primary neoplasms and metastatic lesions in the craniofacial region, particularly gliomas [1]. Initiating radiotherapy soon after surgery can significantly extend the survival of patients with high-grade gliomas [2]. Whole-brain radiotherapy (WBRT), stereotactic radiosurgery (SRS), and SIB-IMRT are common local treatments for brain metastases (BM) [3,4,5]. For patients with BM from lung cancer, WBRT can improve median survival to 3–6 months, with 10–15% of patients surviving beyond a year [6]. It also reduces intracranial tumor size by approximately 60%, improving neurological symptoms and overall prognosis [7,8].

However, radiation-induced neurotoxicity is a significant complication impacting quality of life during and after treatment [9,10]. According to the timing of the occurrence and clinical presentation, radiotherapy-associated cerebral injuries can be divided into three types: acute (occurring within six weeks post-radiotherapy), subacute (occurring between six weeks and six months post-radiotherapy), and late (manifesting months to years’ post-radiotherapy) [11,12,13]. Acute injuries are typified by intracranial hypertension symptoms, such as nausea, vomiting, headache, and lethargy. Late radiation reactions are typically progressive and irreversible, encompassing leukoencephalopathy, radiation-induced necrosis, and various other pathologies [14,15]. Neurocognitive toxicity significantly impacts patients’ quality of life and is a primary concern for clinicians when addressing late-stage radiation toxicity in clinical practice.

Despite considerable research efforts directed at understanding radiation-induced cerebral injury [16,17,18,19], the precise underlying mechanisms remain elusive. Prior investigations primarily emphasized the direct damage incurred by brain parenchymal cells due to ionizing radiation, as well as the cell-to-cell interactions within the cerebral milieu, but these have not led to effective treatments [20,21,22]. Recently, the gut microbiota’s immunomodulatory role in neuroinflammation has garnered significant attention, especially regarding its potential influence on neurocognitive disorders [23,24,25]. While substantial strides have been made in delineating the microbiome–gut–brain axis in other neurocognitive disorders, the domain of radiation-induced cerebral injury warrants further exploration and consolidation.

As quality of life becomes a critical focus in oncology, translational research is essential to develop individualized risk prediction tools and identify mechanisms to reduce the symptom burden of radiation-induced cerebral injury.

## 2. The Risk Factors of Radiation-Induced Brain Injury (RIBI)

**Figure 1:**
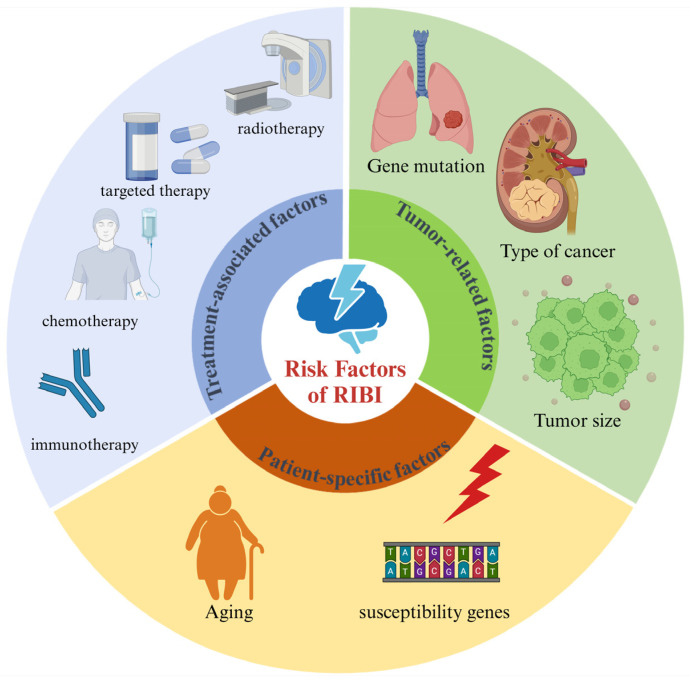
The risk factors of RIBI.

RIBI is influenced by a multitude of factors, including treatment-associated factors, tumor-related factors, and patient-specific factors (Figure 1).

### 2.1. Treatment-Associated Factors

#### 2.1.1. Immunotherapy

Colaco et al. evaluated 180 patients withBM from various cancers, including lung, melanoma, breast, renal, and colorectal, who received SRS in conjunction with cytotoxic chemotherapy, targeted therapy, or immunotherapy. The incidence of radiation necrosis (RN) or tumor-related imaging changes was highest in the immunotherapy group (37.5%), followed by the targeted therapy group (25.0%) and the chemotherapy group (16.9%), indicating a significant correlation between immunotherapy and increased RN rates [26]. Additionally, another retrospective analysis revealed that lung cancer patients with BM who were treated with immune checkpoint inhibitors faced a heightened risk of RN, particularly when administered within three months of cranial radiotherapy [27].

Certain analyses suggest an ambiguous relationship between immunotherapy therapy and RIBI. A meta-analysis of 24 clinical studies found that hypofractionated radiotherapy combined with immunotherapy enhances recurrence-free and overall survival compared to radiotherapy alone without a significant increase in toxicity [28]. In a retrospective study, Eric et al. analyzed 2540 non-small cell lung cancer (NSCLC) patients with BM from 11 institutions, including 395 patients. They reported that the risk of any grade of RN and symptomatic RN post-single SRS and immune checkpoint inhibitors (ICIs) rose when the V12 Gy exceeded 10 cm^3^. However, concurrent immunotherapy and radiosurgery did not significantly elevate this risk, suggesting the importance of minimizing V12 Gy in radiotherapy planning [29].

Hence, caution is imperative when integrating radiotherapy with immunotherapeutic interventions, with a heightened focus on radiographic planning parameters to mitigate potential adverse outcomes. The nuanced integration of these therapies warrants scrupulous monitoring and evaluation to optimize patient safety and treatment efficacy.

#### 2.1.2. Targeted Therapy

A multicenter retrospective study further noted that breast cancer patients with BM had a greater likelihood of symptomatic radiation-induced brain necrosis when treated with concurrent antibody–drug conjugates during cranial radiotherapy [30]. A separate retrospective study covering patients treated with SRS combined with targeted therapy from 2009 to 2022 involved discontinuation of targeted drugs 2–4 days before and resumption 2–4 days after radiotherapy. The targeted therapies encompassed inhibitors targeting ALK/ROS1, EGFR, BRAF, and HER2. The incidence of grade 2 or higher RN was below 6%, with no significant differences across the various targeted drugs [31].

#### 2.1.3. Chemotherapy

Daniel et al. performed a retrospective study involving 149 patients with BM from lung adenocarcinomas who received SRS. Their findings suggested a link between the incidence of imaging-detected necrosis and pemetrexed treatment [32]. A retrospective study of 2843 patients with BM showed no significant difference in the 12-month cumulative incidence of RN with or without systemic therapy. Subgroup analysis indicated that concurrent cytotoxic therapy did not increase the risk of toxicity [33]. Overall, chemotherapy shows a relatively low association with RIBI.

#### 2.1.4. Radiotherapy

The HyTEC study highlights a stratified risk of symptomatic RN associated with different total exposure volumes during 12 Gy single-fraction SRS. The risk increases by 10%, 15%, and 20% at exposure volumes of 5 cm^3^, 10 cm^3^, and >15 cm^3^, respectively, emphasizing the need to limit V12 Gy to a maximum of 10 cm^3^ [5,34,35]. Additionally, a comprehensive retrospective study found that a cumulative dose exceeding 75.7 Gy is a critical factor in RIBI in patients treated for lung cancer BM [27]. While re-radiotherapy contributes to this risk, conclusive evidence remains elusive, with the study yet to reach its endpoint. Data from 388 radiosurgery patients between January 2004 and November 2020 revealed a 15.7% incidence of RN, with a significant increase in risk for those receiving higher radiation doses, indicated by hazard ratios of 1.3 and an amplified risk exceeding 180% at doses between 14 and 20 Gy [36]. A rigorous study led by Professor Chen compared the incidence of grade 5 late complications in patients with locally advanced recurrent nasopharyngeal carcinoma, showing significantly lower severe complication rates in the hyperfractionation group compared to the standard fractionation group [37]. Furthermore, a phase III clinical trial assessing cognitive outcomes in patients with one to three BM treated with SRS alone versus SRS combined with WBRT found reduced cognitive decline at three months in the SRS-only group despite no significant difference in overall survival [38]. These insights augment the nuanced understanding of therapeutic impacts and foster informed decision-making in tailoring individualized treatment paradigms.

### 2.2. Tumor-Related Factors

The type of cancer does not consistently correlate with radiological brain injury across all studies [36,39]. However, some research indicates that patients with kidney and lung adenocarcinoma face a higher risk of RN. In lung adenocarcinoma, patients with EGFR mutations or ALK rearrangements are more prone to RN. In breast cancer, those with estrogen receptor-positive, progesterone receptor-positive, and HER2-amplified cancers exhibit a higher likelihood of RN [40].

Tumor size is also a critical determinant of RIBI. The RTOG 90-05 study examined dose tolerance in SRS for recurrent primary brain tumors or cerebral metastases, specifically assessing grade 4 to 5 neurotoxicity or irreversible grade 3 neurotoxicity occurring within three months post-treatment. The study established dose limits based on tumor size: 24 Gy for tumors ≤2 cm, 18 Gy for tumors measuring 2.1–3 cm, and 15 Gy for tumors measuring 3.1–4 cm [41]. Larger tumors correlated with higher neurotoxicity, emphasizing the importance of individualized dosimetry to enhance therapeutic efficacy while minimizing neurotoxicity. An increase in neurotoxicity grade 3, 4, or 5 was associated with a greater tumor diameter in multivariate analyses. Neurotoxicity was more likely to occur in tumors with diameters of 21 to 40 mm than in those with diameters less than 20 mm [42].

### 2.3. Patient-Specific Factors

#### 2.3.1. Age

Research underscores that the neurotoxic effects of radiation are contingent upon the dose received and the age at exposure [43]. This is especially concerning for children whose developing brains are highly sensitive to radiation, potentially leading to severe long-term neurological issues [44]. Many young survivors of radiation therapy face significant neurocognitive impairments, resulting in motor, intellectual, visual, and psychological dysfunctions [45].

However, understanding radiation effects on the elderly is limited due to their frequent exclusion from clinical trials. Despite this, increasing age poses a substantial risk of cognitive deterioration following WBRT. In this regard, a study by Chan et al. revealed pervasive cognitive decline in elderly patients following WBRT, with all individuals aged 70 and above showing cognitive decline after treatment [46]. This finding emphasizes the necessity of considering patient age when assessing the risks and benefits of radiation therapy and highlights the urgent need for further research into potential mitigating strategies.

#### 2.3.2. Gene

A research team led by Prof. Jia conducted a comprehensive investigation to understand the relationship between genetic variations and disease phenotypes using genome-wide association analysis. They identified a noteworthy mutation site (rs17111237) located in the promoter region of the CEP128 gene on chromosome 14, associated with the development of RIBI in patients suffering from nasopharyngeal carcinoma. This mutation correlated with elevated CEP128 gene expression in individuals with high-risk alleles compared to those with low-risk alleles [47].

Even after adjusting for other clinical risk factors, patients with high-risk genotypes were three times more likely to develop RIBI within five years compared to those with protective genotypes.

In an adjunctive exploration, a meticulous examination of the interconnections between APOE genotypes, baseline protein serum levels, and subsequent radiation-induced neurocognitive declines was undertaken. Contrary to expectations, no substantial correlation was found between APOE genotypes and neurocognitive impairments at the three-month mark. Intriguingly, a potent association emerged between diminished baseline serum concentrations of ApoJ, ApoE, or ApoA proteins and exacerbated neurocognitive debilities. Elevated concentrations of amyloid-beta (Aβ 1-42) further accentuated this discernible trend. Notably, reduced ApoJ levels were significantly linked to cognitive deterioration post-WBRT [48].

These empirical findings imbue a significant augmentation to the comprehension of multifaceted interactions between genetic dispositions, protein serum levels, and radiation therapy, thereby opening avenues for precision-medicine approaches in mitigating cognitive impacts amidst oncological interventions.

## 3. The Mechanism of RIBI

**Figure 2:**
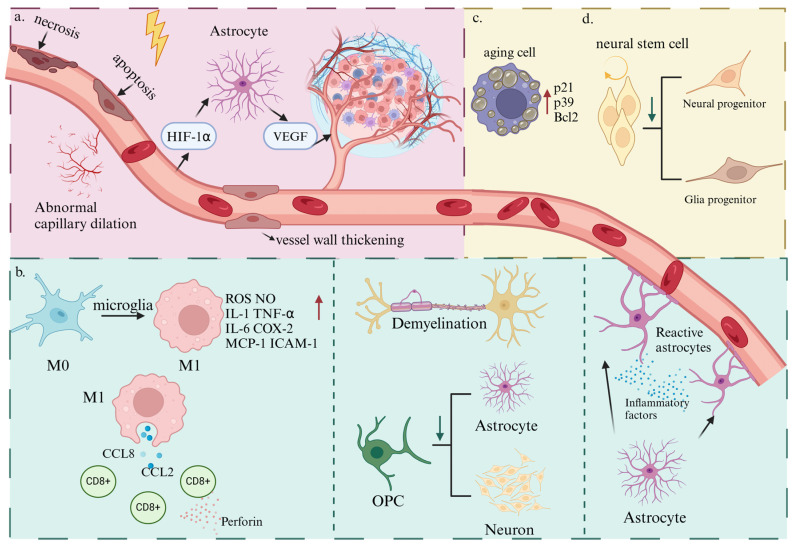
The mechanism of RIBI. Vascular damage and initial RN development (**a**). RN develops within the first 24 h post-radiotherapy due to radiation-induced vascular injury, characterized by endothelial apoptosis and necrosis, followed by capillary collapse and basement membrane thickening. This damage triggers HIF-1α expression, leading to VEGF secretion by glial cells and abnormal angiogenesis. Glial cell dysfunction (**b**). Radiotherapy induces glial cell activation and neuroinflammation, resulting in astrocyte proliferation and glial scar formation, impaired oligodendrocyte differentiation, and demyelination. Activated microglia release pro-inflammatory cytokines (IL-1, TNF-α, COX-2) and recruit CD8+ T cells, which secrete cytotoxic factors and worsen inflammation. Cellular senescence (**c**). Radiation induces premature senescence in endothelial and glial cells, marked by increased expression of senescence-associated genes (P21, P39), contributing to radiation-induced tissue aging and delayed RN. Neurogenesis dysfunction (**d**). Ionizing radiation reduces the self-renewal and differentiation capacity of neural stem cells, leading to decreased neurogenesis and long-term neurocognitive toxicity.

RIBI is presently conceptualized as a multistage phenomenon, implicating a plethora of cellular entities. The cardinal mechanisms implicated encompass the following: (1) Vascular insult leading to a compromise in brain perfusion, (2) damage to glial cells, pivotal in maintaining homeostasis, instigating neuroinflammation, (3) induction of cellular senescence processes contributing to an aging phenotype, and (4) dysregulation of neural stem cell functionality, disrupting the equilibrium of neural regeneration and repair (Figure 2).

### 3.1. Vascular Damage

RIBI initiates with a biphasic pattern of vascular alterations. The acute phase typically manifests within 24 h post-exposure, primarily driven by radiation-induced endothelial cell apoptosis [49]. The chronic phase, spanning several months, is marked by capillary collapse, thickening of the basement membrane, and endothelial clonogenic activity ceases [50,51]. The loss of endothelial cells may lead to vascular leakage, potentially inciting cognitive decline [52].

Irradiation induces apoptosis and senescence in microvascular endothelial cells [53]. Advanced vascular changes, including capillary and microvascular dilation and vessel wall thickening, can precipitate ischemic stroke, cerebral microbleeds, and small vessel occlusion, resulting in secondary white matter necrosis and subsequent cognitive impairment [54,55].

Radiation impacts the vascular tissue surrounding the tumor, damaging vascular tissue and impeding oxygen diffusion, which induces tissue hypoxia and increases hypoxia-inducible factor (HIF-1α) expression [56]. Elevated levels of HIF-1α expression stimulate reactive astrocytes to secrete the pro-angiogenic factor vascular endothelial growth factor (VEGF) [57,58], leading to the formation of aberrant neovascularization [59,60,61]. These newly formed structures are characteristically disordered and fragile, with high permeability that promotes exudation from surrounding tissues, ultimately inducing cerebral edema. The resultant edema precipitates local tissue ischemia and hypoxia, ultimately leading to radiation-induced cerebral necrosis.

### 3.2. Aberrant Activation or Damage of Glial Cells

#### 3.2.1. Microglia Activation

Microglia, the resident immune cells in the brain, play multiple roles in brain development and homeostasis, including immune surveillance, inflammation regulation, clearance and phagocytosis, neurotrophic and metabolic support, as well as neuroprotection and repair [62,63,64]. Physiologically, microglia remain quiescent [65]. However, radiation exposure prompts microglia to perceive alterations in the surrounding microenvironment and respond accordingly. Sustained activation of microglia can lead to chronic neuroinflammation and cognitive impairments during the late stages of RIBI. Recent advancements in neuroimmunology have elucidated that microglial activation involves a critical phenotypic shift from an anti-inflammatory M2 state towards a pro-inflammatory M1 state [66,67,68]. This transition is characterized by increased production of reactive oxygen species (ROS) and nitric oxide (NO), along with elevated levels of key inflammatory mediators, including interleukin-1 (IL-1), tumor necrosis factor-alpha (TNF-α), interleukin-6 (IL-6), cyclooxygenase-2 (COX-2), monocyte chemoattractant protein-1 (MCP-1), and intercellular adhesion molecule 1 (ICAM-1) [69,70,71,72]. The continued secretion of pro-inflammatory factors by activated microglia maintains an inflammatory state within the brain microenvironment, contributing to the death of neurons and progenitor cells, thus establishing a vicious cycle of microglial activation, inflammatory factor release, and neuronal death [73,74,75]. Rodent studies have demonstrated that, following a single high-dose irradiation event, there is a sustained elevation in both activated microglia and TNF-α levels, persisting for a minimum duration of six months [76,77]. Furthermore, Tang et al. reported that in the aftermath of radiation-induced brain stimulation or cerebral ischemia, microglia inflict secondary brain damage by secreting chemokines CCL2 and CCL8. This process attracts peripheral CD8+ T cells to infiltrate, leading to the release of cytotoxic factors such as perforin and granzyme [78].

#### 3.2.2. Reactive Astrogliosis

Astrocytes, a significant component of the brain cells, perform crucial roles in central nervous system (CNS) maturation, neuron support, homeostasis maintenance, and neurotransmitter regulation [79,80]. Under physiological conditions, astrocytes present as small cells with short processes. However, during pathophysiological events such as CNS injury, inflammation, or exposure to toxins, astrocytes transition from a naive state to a reactive phenotype, termed astrocytic reactive hyperplasia [81].

Depending on the severity of neural tissue injury, astrocytes undergo activation and proliferate throughout the affected region, a process known as reactive astrocytosis. This leads to the eventual formation of glial scarring [82,83]. Reactive astrocytes exhibit increased proliferation, cellular hypertrophy, upregulation of the intermediate filament glial fibrillary acidic protein (GFAP), and augmented secretion of various pro-inflammatory mediators, including cyclooxygenase and a novel variant, intercellular adhesion molecule-29 (ICAM-29) [84,85,86]. This panoply of pro-inflammatory mediators underpins various inflammatory and remodeling processes. Zhou et al. demonstrated that X-ray irradiation could directly activate astrocytes in vitro, leading to reactive proliferative hypertrophy, increased GFAP expression, and elevated intracellular VEGF expression, potentially leading to radiation brain injury [87].

#### 3.2.3. Oligodendrocytes Damage

Oligodendrocytes, constituting approximately 45% of the total glial cell population, are a dominant cell type in the human white matter [88]. These cells have been shown in several studies to support axonal metabolism [89,90] and regulate the behavior of neural networks [91,92]. Crucially, oligodendrocytes are responsible for the formation and maintenance of myelin sheaths in the CNS, making them vital in RIBI. Demyelination, a key feature of delayed radiation injury, is significantly influenced by oligodendrocytes. Compared to other glial cells like microglia, oligodendrocytes have exhibited a markedly increased sensitivity to radiation [93,94]. Ionizing radiation can directly trigger oligodendrocyte apoptosis [95]. Radiation-induced oxidative stress hampers oligodendrocyte maturation [96] and is linked to demyelinating neuroinflammation [97]. Furthermore, Oligodendrocyte precursor cells (OPCs) are also vulnerable to the effects of ionizing radiation, which could result in diminished differentiation capabilities into astrocytes and neurons [98]. Therefore, radiation-induced damage to OPCs can indirectly affect the function of other CNS cell types [99]. Oligodendrocyte damage and subsequent demyelination are causal factors in delayed RN [100].

### 3.3. Radiation-Induced Aging

It has been suggested that radiation treatment contributes to cellular senescence, a condition of irreversible cell cycle cessation, which is seen as a crucial element in tissue aging and degenerative ailments [101]. This phenomenon is marked by the increased expression of senescence-associated β-galactosidase (SA-β-Gal) and the upregulation of senescence-specific genes such as p2, p39, and Bcl2, which have been detected in macrophages derived from bone marrow after being exposed to radiation [102]. In vitro investigations reveal that radiation exposure induces a stress-induced premature senescent phenotype in endothelial cells, eliciting the expression of P16 and resulting in permanent cell cycle arrest. Gamma irradiation-induced damage to cerebral microvasculature accelerates aging in healthy tissues and contributes to cognitive decline in 50% of tumor patients undergoing radiotherapy [103]. Furthermore, irradiated microglia also exhibited upregulated expression of the key senescence markers, SA-β-Gal and p16INK4a; even a month after exposure, these phenomena may be associated with radiation-induced oxidative stress, autophagy, telomere attrition, and mitochondrial dysfunction [104]. In in vitro primary cell cultures, irradiated astrocytes did not transition to reactive astrocytes [105], but indicators of the aging phenotype were observed. An investigation revealed a notable increase in astrocyte senescence within irradiated human brain tissues [106]. Cellular senescence manifests as tissue-level aging. Nikhil Rammohan and colleagues examined 4220 individuals, including 4148 healthy participants and 72 patients exposed to an average radiation dosage of 30 Gy. The findings indicated that the irradiated patients exhibited brain and substructure changes consistent with accelerated aging compared to healthy controls. In a subset analysis, the hippocampus exhibited an accelerated aging rate of 8.88 times following conventional WBRT compared to hippocampus-sparing WBRT protocols [107]. Remes et al. followed childhood brain tumor survivors who had undergone radiation therapy for 20 years. The study found that their rates of ischemic infarction, microbleed, and lacunar infarction were comparable to or exceeded those of the general population aged over 70, supporting the theory that radiation accelerates cerebrovascular aging [106]. Senescence leads to delayed RIBI. This underscores the long-term consequences of radiation exposure in young patients and highlights potential therapeutic targets to mitigate these effects.

### 3.4. Neurogenesis Dysfunction

Radiation-induced neurocognitive damage is marked by the activation of microglia in the dentate gyrus and apoptosis of myeloproliferative cells in the hippocampus’s subgranular zone, a critical area for lifelong learning and memory due to its neurogenic capacity [108,109]. Neural stem cells, crucial to neurogenesis, are adversely impacted by ionizing radiation, which affects their proliferation, maintenance of the cell cycle, and stemness [110]. Moreover, radiation impedes the specialization of neural stem cells and modifies the manifestation of proteins associated with the formation of new neurons. Post-exposure, a sustained reduction in neurogenesis in the hippocampus’s subgranular region is typically observed [111,112,113,114,115,116]. These disruptions can cause chronic, irreversible cognitive deficits and dementia. Immediate effects can occur, but it is the delayed impacts that often result in long-term cognitive impairments and dementia [88,117].

## 4. Preventive and Therapeutic Measures

Current strategies for preventing and treating RIBI include advancements in radiotherapy techniques, pharmacological interventions, and physical therapies (Table 1).

**Preventive Measures**

### 4.1. Hippocampal Avoidance Radiotherapy and Hypofractionated Stereotactic Radiotherapy

The hippocampus is crucial for memory formation, with its dentate gyrus cells being especially susceptible to radiation damage during radiotherapy for nasopharyngeal cancer [118]. The severity of hippocampal damage is predictive of cognitive dysfunction (CD) duration [119], highlighting the importance of hippocampal neurogenesis in radiotherapy-induced cognitive impairment [112,120]. Thus, strategies to protect the hippocampus are vital to mitigate RIBI.

A phase III multicenter clinical trial showed that hippocampal-avoidance prophylactic cranial irradiation (HA-PCI) reduced hippocampal atrophy in small-cell lung cancer patients at 4- and 12-months post-treatment. However, the trial did not find a significant correlation between hippocampal atrophy and memory decline as assessed by the HVLT-R, leaving the benefits of HA-PCI debated [121]. A separate investigation revealed a significant reduction in the occurrence of RN when treating medium-sized BM with hypofractionated SRS compared to single-fraction SRS (2.5–3 cm) [122].

**Table 1:** Treatment measures.

**Treatment**	**Medications**	**Mechanisms and Effects**
anti-VEGF [123,124,125,126,127]	Bevacizumab	Inhibiting VEGF to reduce angiogenesis and regulate vascular permeability
Corticosteroids [128,129,130]	Methylprednisolone, prednisolone, dexamethasone	Alleviating vascular edema symptoms associated with radiation-induced brain injury
PPAR agonists (PPARs) [131,132,133,134,135,136]	Fenofibrate, pioglitazone	Activating neuroprotective and anti-inflammatory pathways to mitigate radiation-induced cognitive impairment and delay cognitive decline
NMDA receptor antagonists [137,138,139,140]	Memantine, donepezil	Non-competitively blocking NMDA receptors to prevent glutamate-induced excitotoxicity and improve CD caused by radiation
Thalidomide [141,142,143]	Thalidomide	Enhancing vascular stability and maturation to improve cerebral blood flow and cognitive function
Pentoxifylline/Vitamin E [144,145,146,147]	Pentoxifylline/Vitamin E	Improving microcirculation with antioxidant effects to reduce radiation-induced brain necrosis
Boswellia Serrata [148,149,150,151,152,153,154]	Boswellia Serrata	Reducing pro-inflammatory factors to alleviate brain edema and radiation-induced damage
Hyperbaric Oxygen Therapy [155,156,157,158]		Stimulating angiogenesis to restore blood supply, reduce inflammation cell recruitment, and promote tissue repair

### 4.2. Medications

#### 4.2.1. Bevacizumab

Bevacizumab, a recombinant human monoclonal antibody, binds to VEGF, preventing its interaction with endothelial cell surface receptors Flt-1 and KDR [123,124]. This mechanism reduces blood vessel formation, regulates vascular permeability, and decreases brain edema caused by cerebral necrosis, effectively treating the condition [125]. While many studies highlight the positive short-term impact of bevacizumab in treating RIBI [126], debate persists regarding the optimal dosage, duration, and timing. It is crucial to understand that bevacizumab primarily targets the blood vessels surrounding the necrotic area, modulating cerebral edema caused by newly sprouted blood vessels, but it does not directly address the necrosis itself. Consequently, local ischemia and hypoxia persist. If treatment with bevacizumab is halted, the expression of HIF-1α might surge again in the tissue surrounding the necrotic area, triggering a vicious cycle that may eventually lead to a recurrence of cerebral necrosis [127].

#### 4.2.2. Glucocorticoids

Traditional treatment for RIBI typically involves glucocorticoids [128]. Corticosteroids are the first-line treatment for RIBI, providing rapid symptom relief by reducing cerebral edema and inflammation [129]. However, long-term use may lead to side effects such as steroid dependence, gastrointestinal bleeding, and infections. Balancing symptom relief with potential adverse effects is crucial in clinical practice. Studies on patients with RIBI from nasopharyngeal carcinoma revealed no significant differences in symptom improvement or cognitive function between low-dose and high-dose methylprednisolone groups, while the low-dose group experienced fewer grade 3 adverse events [130]. Appropriate control of corticosteroid dosage and duration effectively alleviates symptoms while minimizing the risk of side effects.

#### 4.2.3. Peroxisome Proliferator-Activated Receptors (PPARs)

PPAR, a member of the steroid/thyroid hormone superfamily of nuclear receptors, is recognized for its pivotal role in activating neuroprotective and anti-inflammatory pathways within the CNS [131,132]. Current evidence underscores the significance of all PPAR subtypes during both developmental stages and adulthood within the cerebral milieu [132]. In vitro experiments employing the PPAR agonist GW7647 and fenofibrate notably suppressed the expression of inflammatory cytokines in microglial cells following ionizing radiation exposure [133]. Animal studies have shown that PPAR agonists can decrease the long-term cognitive impacts of radiation [134]. In one study, mice receiving 10 Gy of WBRT and treated with fenofibrate exhibited increased hippocampal neuron numbers and decreased microglial activation [135]. Preliminary results from a Phase I clinical trial (NCT01151670) indicate that daily administration of 45 mg pioglitazone during radiotherapy and for six months afterward significantly improved learning, calculation, and memory abilities in patients while confirming its safety in non-diabetic individuals [136].

#### 4.2.4. Memantine Hydrochloride

The N-methyl-d-aspartate (NMDA) receptors are pivotal in synaptic genesis, maturation, plasticity, neural network activity, and cognitive functions [137]. Memantine Hydrochloride is an NMDA receptor antagonist used in the treatment of moderate to severe vascular dementia and Alzheimer’s disease (AD) [138]. Yohei Hokama and colleagues first discovered in animal models that combining memantine with hyperbaric oxygen therapy during radiotherapy can repair white matter damage and promote neurogenesis, thereby improving anxiety-like behaviors and cognitive impairments in mice. In subsequent clinical trials, neuroimaging and neuropsychological assessments similarly confirmed that memantine can enhance higher cognitive functions in patients undergoing cranial radiotherapy [139]. In a large-scale randomized, double-blind, placebo-controlled trial, memantine did not significantly improve memory retention compared to placebo in patients with BM undergoing WBRT. However, memantine extended the time to cognitive decline and demonstrated favorable tolerability [140].

#### 4.2.5. Thalidomide

Radiation-induced vascular disruption is a key mechanism in the pathogenesis of RIBI. Studies have shown that thalidomide can inhibit vascular instability in animal models and normalize tumor vasculature by enhancing vascular maturity, pericyte coverage, and endothelial junction integrity [141]. Thalidomide has demonstrated good efficacy in treating small intestine vascular malformations [142]. A Phase II clinical study revealed that thalidomide could improve cerebral perfusion in an RIBI mouse model by rescuing pericyte function, thereby enhancing cognitive function. In concurrent human trials, 43.1% of patients showed clinical symptom improvement, and 62.1% had increased cognitive scores, suggesting thalidomide’s therapeutic potential in treating radiation-induced cerebrovascular injury [143].

#### 4.2.6. Pentoxifylline/Vitamin E

Pentoxifylline (Ptx), a dimethylxanthine derivative, enhances microcirculation in ischemic tissues and increases oxygen delivery to organs, while vitamin E(VitE), as an antioxidant, scavenges free radicals and inhibits lipid oxidation. The combination of Ptx and VitE has been supported by multiple studies for mitigating radiation-induced side effects, such as pneumonitis [144] and fibrosis [145]. In preclinical studies of RIBI, this combination reduced VEGF and HIF-1α levels in intracranial vasculature, alleviating RN in rat models [146]. Research by Jimmy S. Patel et al. demonstrated that Ptx combined with VitE improved MRI findings in nearly half of patients with RN following SRS, with a median response time of 3.17 months and minimal side effects [147].

#### 4.2.7. Boswellia Serrata

Boswellia serrata (Indian frankincense extract) exhibits significant anti-inflammatory effects by inhibiting pro-inflammatory factors and 5-lipoxygenase (5-LO) synthesis [148], showing efficacy in conditions such as asthma, arthritis, and colitis [149]. Preclinical studies suggest Boswellia serrata extract (BSE) protects dopaminergic neurons from rotenone-induced neurotoxicity by activating the AMPK pathway [150]. Additionally, BSE reduces fipronil-induced neurotoxicity by inhibiting oxidative stress, inflammation, and apoptosis pathways, alleviating neuronal necrosis and astrocytic proliferation [151]. Furthermore, Boswellia has been demonstrated to reduce cerebral edema [152].

A preliminary clinical study involving 50 patients with CTCAE v5.0 grade 1–3 RN showed a response rate exceeding 50% with daily BSE doses of 4.2–4.5 g. Among them, 39 patients continued treatment until follow-up or death [153]. Another study reported significant neurological and imaging improvements in three patients with RN refractory to corticosteroids and Ptx/VitE after supplementation with 5-Loxin (Boswellia serrata). These patients successfully discontinued steroids without adverse effects [154].

### 4.3. Physical Therapies

#### Hyperbaric Oxygen Therapy (HBOT)

Increasing oxygen concentration stimulates angiogenesis, restores blood supply to necrotic lesions, and promotes healing. HBOT reduces radiation-induced damage by inhibiting neutrophil and macrophage recruitment and suppressing inflammatory cytokine synthesis [155,156]. A case report documented significant clinical and imaging improvements in a 5-year-old post-surgical brain tumor patient who developed RN 11 months after radiotherapy and underwent HBOT [157]. Another study involving 13 RN patients treated with HBOT showed sustained symptom improvement in most cases, with otologic complications as the primary side effect [158]. Currently, most evidence for HBOT comes from case reports, and further clinical studies are needed to validate its efficacy.

## 5. Gut–Brain Axis

**Figure 3:**
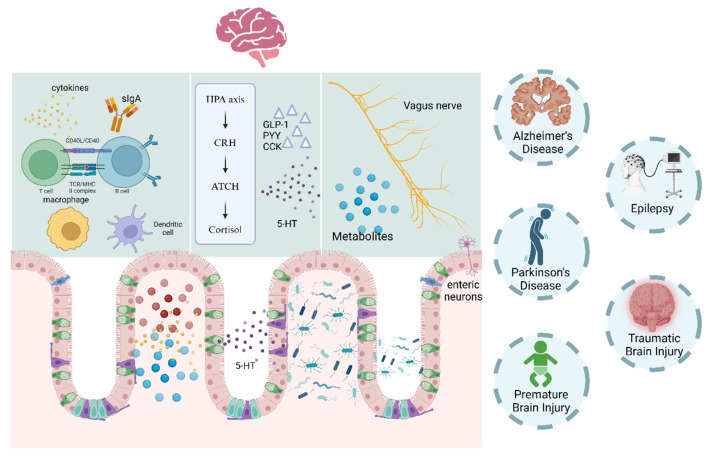
The gut–brain axis and relevant disease.

The gut microbiota, comprising thousands of bacterial species, begins to form at birth through exposure to maternal microbes [159]. There is a complex interaction network between the gut microbiota and the host, involving pathways such as the gut–brain axis, gut–liver axis, and gut–lung axis. The gut–brain axis, a bidirectional communication network between the gut and brain, is a key pathway linking gut microbiota to neurological function [160,161,162,163]. The CNS regulates gut motility and digestion [164,165], while gut microbes influence neurodevelopment and inflammation [166]. Studies in germ-free mice reveal the microbiota’s role in processes like neurogenesis, myelination, and maturation [167]. Maternal microbiota deficiencies can impair embryonic axonal development, affecting offspring’s sensory behaviors [168]. The gut microbiota interacts with the nervous system through neural, immune, and endocrine pathways (Figure 3). Focusing on the gut–brain axis helps identify new therapeutic targets for neurological disorders.

The gut–brain axis interacts through three primary pathways: neural, immune, and endocrine. Cytokines, metabolic products, and neurotransmitters also participate, forming a complex network of interactions.

## 6. Interaction Pathway Between Gut Microbiota and Brain

### 6.1. The Neural Pathway

Gut bacteria impact the nervous system by producing neurotransmitters, neurotrophic factors, and metabolic products. The vagus nerve (VN) and the enteric nervous system (ENS) are direct pathways connecting the gut and the brain [169]. Microbial products, hormones regulated by the microbiota, and immune mediators dependent on microbiota have the ability to directly engage with intrinsic enteric neurons as well as vagal and spinal afferent nerves innervating the gut [166]. Additionally, the gut microbiota probably can indirectly interact with mechanosensory, which sense mechanical signals and transmit them via the VN to the brain, forming distinct mechanosensory circuits that regulate neural development [170,171]. Bacteroides fragilis and Lactobacillus rhamnosus have been shown to stimulate intestinal afferent neurons in vitro [172]. Research by Bercik demonstrated that Bifidobacterium longum NCC3001 alleviates anxiety behavior in mice with chronic colitis and elevates brain-derived neurotrophic factor mRNA expression in the hippocampus. This effect relies on the VN, as it vanishes after vagotomy [173].

Certain gut microbes directly influence the nervous system through neuroactive metabolites. For example, specific Bifidobacterium strains produce GABA, an inhibitory neurotransmitter that modulates neuronal excitability and anxiety [174]. Metabolites of Edwardsiella tarda, such as indole, activate TRPA1 channels in entero-chromaffin cells, triggering serotonin release, which excites gut neurons, enhances motility, and modulates the CNS via vagus nerve stimulation [175]. In antibiotic-treated mice, elevated monoamine oxidase levels increase dopamine degradation, reducing dopamine responsiveness. Conversely, germ-free mice receiving fecal transplants from energetic donors exhibit significantly elevated dopamine levels [176,177]. Dysbiosis, such as that induced by high-fat, high-sugar diets, increases acetylcholinesterase (AChE) expression in the brain, disrupting cholinergic signaling and impairing memory, learning, and attention [178]. Short-chain fatty acids (SCFAs) target GPR41/FFAR3 receptors expressed in sympathetic and vagal ganglia, suggesting direct neuronal activation [179]. Bile acids, common microbial metabolites, interact with FXR and TGR5 receptors expressed in brain neurons, indicating their potential role in gut–brain axis signaling [180].

### 6.2. The Immune Pathway

The gut microbiota interacts with the immune system through itself and its metabolites, primarily mediated by microglia, influencing the CNS. Dysbiosis can impair the intestinal barrier [181]. Bacteria can secrete immune stimulators such as lipopolysaccharides (LPS) and peptidoglycans into the circulation, enabling their entry into the brain. Gut mucosal pattern recognition receptors, like Toll-like receptors (TLRs), recognize microbial molecules such as LPS, which activate immune cells (e.g., dendritic cells, neutrophils, macrophages) and lead to the production of pro-inflammatory cytokines [182].

Certain cytokines can cross the blood–brain barrier directly, altering the inflammatory state of the CNS. For example, elevated levels of IL-6 in the brain can disrupt the balance of excitatory/inhibitory synaptic transmission and lead to autism spectrum disorder behaviors [183]. Additionally, gut microbiota influence brain function by regulating intracranial immune cells like microglia. During aging, increased Ruminococcaceae produce metabolites like isovaleric acid (IAA), regulating pro-inflammatory and pro-apoptotic factor S100A8 expression, mediating microglial apoptosis, and contributing to cognitive decline [184]. SCFAs regulate C3 signaling in the CNS and may contribute to C3/CR3-dependent microglial synaptic clearance [185]. Acetate promotes microglial maturation and metabolic homeostasis by stabilizing mitochondria, playing a role in the progression of neurodegenerative diseases [186]. Additionally, local CNS immune cells can be “trained” by the gut microbiota, which induces systemic interferon-γ secretion. This cytokine reaches natural killer cells in the meninges, fostering the development of neuroimmune-regulatory astrocytes expressing LAMP1 and TRAIL [187]. Besides the endocrine signaling of immune factors across the gut–brain axis, immune cells within the gut can directly modulate neuroimmune balance and inflammatory responses in the brain. Gut antigens stimulate B cells to differentiate into IgA+ plasma cells, which control gut microbiota homeostasis. In autoimmune diseases of the nervous system, IgA+ plasma cells are recruited to the brain and spinal cord to mitigate neuroinflammation [188]. Conversely, brain inflammation can influence the composition of the gut microbiome, initiating a detrimental cycle where gut dysbiosis intensifies neuroimmune responses, exacerbating brain pathology and behavioral changes.

### 6.3. The Endocrine Pathway

The gut, as the body’s largest endocrine organ, contains epithelial enteroendocrine cells (EECs) that are crucial for gut–brain endocrine communication. These cells produce various hormones, including glucagon-like peptide 1 (GLP-1), peptide YY (PYY), and serotonin (5-HT) [189]. Due to the blood–brain barrier, gut-derived 5-HT does not directly affect the CNS but indirectly modulates brain function via immune pathways. For example, 5-HT binds to receptors on T cells, inhibiting CD8 T cells from releasing IL-17 and IFN-γ [190]. GLP-1 influences brain function by enhancing hippocampal neuroplasticity and providing neuroprotection, with its receptors widely distributed in the CNS and other tissues [191]. PYY acts through the circulatory system on appetite-stimulating neurons in the brain, inhibiting their activity, regulating the hypothalamus, increasing satiety, and reducing food intake [192].

In the nervous system, a stressful environment can activate the hypothalamic–pituitary–adrenal (HPA) axis, promoting hypothalamic neurons to secrete corticotropin-releasing hormone (CRH) into the brain or portal circulation. This triggers the release of adrenocorticotropic hormone (ACTH), which in turn stimulates the synthesis and secretion of cortisol. Cortisol regulates the integrity of the gut barrier through neuroimmune signaling. Stress hormones, immune signals, and central neurotransmitters can stimulate neurons within the ENS and the vagal afferent pathway. This neuronal activation leads to alterations in the gut environment and shifts in the microbiota composition [193]. Studies indicate that microbiota can enhance social behavior in mice under high social stress by suppressing HPA axis activation and reducing corticosterone levels. This effect is attenuated by depleting the gut microbiota with antibiotics and can be reversed by adrenalectomy, glucocorticoid receptor antagonists, and cortisol synthesis inhibitors [194].

## 7. Gut Microbiota in Neurodevelopmental Disorders

### 7.1. Alzheimer’s Disease

The etiology of AD remains unclear, with primary hypotheses including neuroinflammation, Tau protein phosphorylation, and Aβ protein deposition [195]. Studies have shown that the gut microbiota diversity in AD patients differs from that of healthy individuals [196]. Reports indicate an increased abundance of Actinobacteria and Firmicutes in AD patients, with some of these bacterial groups playing crucial roles in neuroinflammation and neurodegenerative diseases [197]. An RNA sequencing analysis of the brains of specific pathogen-free and germ-free mice has revealed several signaling pathways involved in AD pathology. For instance, compared to GF mice, SPF mice exhibited alterations in the insulin/IGF-1 and related downstream pathways, as well as the C/EBPβ/AEP pathway. Modulating the gut microbiota composition may improve the progression of AD [198]. Research involving long-term treatment of transgenic mice with a mix of antibiotics has indicated reduced aggregation of microglia and astrocytes around amyloid plaques in the hippocampus, as well as decreased insoluble amyloid beta plaques [199]. Holtzman et al. also found that antibiotics significantly altered microglial and astrocytic gene expression and morphology, reducing phosphorylated tau proteins thus preventing tau-associated neurodegenerative lesions [200]. The use of probiotic supplements, such as Bifidobacterium longum and Lactobacillus acidophilus, has been shown to delay the progression of AD in male APP/PS1 transgenic mice [201].

Metabolites of the gut microbiota also play a significant role in AD. For example, lysophosphatidylcholine, a metabolite of Bacteroides ovatus, alleviates AD by inhibiting neuronal ferroptosis [202]. Studies have shown that the levels of SCFAs in the brains of five × FAD mice are positively correlated with microglial activation, and the levels of isobutyric acid in the feces, serum, and brain tissues of Tg-M mice are higher than those in wild-type mice, which is closely associated with neuroinflammation and cognitive decline [203]. Butyrate suppresses microglial activation, reduces pro-inflammatory cytokines in APP/PS1 mice, and inhibits Aβ-induced NF-κB signaling via COX-2 and CD11b downregulation [204]. Trimethylamine N-oxide (TMAO) accelerates Aβ and tau pathology by activating PI3K/AKT/mTOR signaling [205]. A high-tryptophan diet effectively alleviates gut microbiota dysbiosis in APP/PS1 mice and inhibits microglial reactivity by activating the central AHR signaling pathway and suppressing NF-κB signaling [206]. TUDCA treatment has been shown to inhibit Aβ deposition and neuroglial activation in APP/PS1 mice and A7-Tg mice fed a high-fat diet [207]. These findings suggest the microbiota plays a part in controlling crucial molecular elements of AD.

### 7.2. Parkinson’s Disease

PD is a neurodegenerative disorder with prominent gastrointestinal manifestations. It is now accepted that α-synuclein is a central player in the development of PD. Lewy bodies, which contain α-Syn, initially appear in the gut’s enteric neurons [208], with the VN serving as a crucial conduit between the enteric nervous system and the CNS, facilitating the spread of α-Syn from the gut to the brain [209]. The transmission of Lewy’s pathology from the gut to the brain is impeded when vagotomy is performed in mice models of PD. Additionally, individuals who undergo surgical removal of their VN experience a notable decrease in the likelihood of developing PD [210]. Gut bacteria from Parkinson’s patients exacerbate motor and gastrointestinal dysfunctions, microglial activation, and α-Syn pathology in transgenic mice. Antibiotics mitigate these effects, while fecal transplants from Parkinson’s patients worsen motor impairments [211]. The gut microbiota in PD patients lacks SCFA-producing bacteria, such as Faecalibacterium, which is known for its anti-inflammatory properties [212]. Gut microbiota from PD patients disrupts Th17 immune homeostasis in the small intestinal mucosa, leading to gut inflammation and barrier dysfunction. This results in the loss of midbrain tyrosine hydroxylase-positive cells and motor impairments in mice [213]. Another key neuropathological hallmark of PD is the loss of dopaminergic neurons in the SN. In Pink−/− mice with Gram-negative bacterial gut infections, there is a marked reduction in striatal dopamine synapse density, which can be reversed by levodopa treatment, suggesting gut infections can trigger Parkinsonian symptoms [214]. An innovative drug targeting the gut–brain axis, GV-971, significantly reduces α-synuclein accumulation and aggregation in mouse models, mitigating α-synuclein-induced neuronal damage and restoring extracellular vesicle release [215].

### 7.3. Premature Brain Injury

Premature infants are notably susceptible to perinatal white matter injury, characterized by an altered gut microbiome profile. Notably, there is a decline in beneficial microbes such as Bifidobacterium and Lactobacillus, accompanied by a rise in potential opportunistic pathogens, including Escherichia coli, Enterococcus, and Klebsiella [216]. This microbial shift is significant, as Escherichia coli, a key contributor to the gut’s LPS load, is implicated in stimulating the blood–brain barrier and activating microglia, thereby inducing hippocampal neuron apoptosis and amyloid-beta deposition in the thalamus [217], which are critical factors in the onset of cognitive impairments in these infants. Group B Streptococcus (GBS) frequently colonizes the neonatal gut. Immature gut microbiota reduces resistance to GBS colonization, promotes intestinal barrier leakage, and increases systemic bacterial load, facilitating GBS translocation and impairing CNS development [218].

Further, a murine model for CD in preterm neonates reveals that cognitive deficits correlate with diminished gut microbiota diversity and Proteo-bacteria proliferation, coupled with a decrease in total SCFAs and acetate levels [219]. In a seminal study by David Seki et al., the gut microbiome, immune response, and neurophysiological development of 60 preterm infants under standard hospital care were meticulously analyzed. The study establishes a link between gastrointestinal overgrowth of Klebsiella, heightened levels of specific immune cells, and the progression of brain injury in these infants. This underscores the pivotal role of abnormal development in the gut–microbiome–immune–brain axis in exacerbating cerebral damage in extremely preterm infants [220].

### 7.4. Epilepsy

Epilepsy is a common disease that affects people of all ages, races, and geographical areas [221]. Epilepsy patients often present with gastrointestinal symptoms, and individuals with inflammatory bowel disease have a higher susceptibility to epilepsy [222]. At the core of epileptic seizures is an aberrant and excessive discharge of cortical neurons, underscoring a complex neurophysiological process [223]. Currently, available anti-epileptic drugs are effective for about two-thirds of patients with epilepsy. Recent scientific discourse has pivoted towards the intriguing correlation between gut microbiota and epilepsy. A study highlighted a marked increase in the α-diversity of gut microbiota in epileptic patients alongside distinct compositional shifts. Compared with healthy individuals, the epilepsy group contained a higher proportion of Bifidobacterium, Streptococcus, Akkermansia, Enterococcus, Prevotella, Veillonella, Roseburia, and Clostridium quartum, while the proportions of Bacteroides and Faecalibacterium were lower [224]. Conversely, Medel-Matus et al. provided compelling evidence that gut microbiota modulation in normal rats could mitigate epileptic responses in stress-induced models [225]. The microbial metabolite butyrate mitigates oxidative stress and neuronal apoptosis in brain tissue by activating the Keap1/Nrf2/HO-1 pathway, thereby increasing seizure thresholds and reducing seizure intensity [226]. Levels of IL-17 in cerebrospinal fluid CSF and peripheral blood are significantly elevated in epilepsy patients compared to controls, correlating closely with seizure frequency and severity [227]. IL-17, produced by Th17 cells, could be modulated by the phylum Bacteroidetes [228]. Another study showed that 50.28% of patients with drug-resistant epilepsy reduced the frequency of epileptic seizures by 9% or more after treatment with probiotics [229].

### 7.5. Traumatic Brain Injury (TBI)

Recent preclinical studies have shed light on the complex interplay between TBI and the gut microbiota, demonstrating that TBI profoundly impacts both gut function and microbiota composition. In a rat model of moderate TBI, researchers employed fecal 16S rRNA sequencing to identify changes in the gut microbiota, with changes evident as early as two days post-injury [230]. Within a week following TBI, a significant reduction in beneficial bacteria such as Bifidobacterium and Lactobacillus is frequently observed, along with a sharp increase in opportunistic pathogens like Escherichia coli and Clostridium, which compromise blood–brain barrier integrity and exacerbate neuroinflammation [231]. Another critical preclinical study focusing on diffuse brain injury indicated that TBI-induced microbial ecological disruption, in conjunction with gastrointestinal dysfunction and changes in bile acid composition, were crucial mediators of gut microbiome–host interactions [232]. Gut microbiota depletion using an antibiotic cocktail has been shown to improve neurological outcomes and reduce lesion volume in TBI [233].

Microglial changes after TBI were found to be time-dependent and long-lasting, affected by microbial ecological disruption up to three months post-injury [234,235]. Administration of antibiotics seven days post-TBI increased microglial activation markers, including toll-like receptor 4 and major histocompatibility complex II [236]. SCFAs have been demonstrated to inhibit microglial activation, reduce pro-inflammatory cytokine production, and promote regulatory T cell differentiation in experimental TBI models, thereby suppressing immune responses and preventing excessive inflammation [237]. In addition, disruption of the gut microbial ecology led to an acute decrease in peripheral blood monocyte circulation and infiltration into the brain parenchyma. Although no significant changes in T cell infiltration were observed three days post-injury, the introduction of lymphocytes and monocytes into the hippocampus was inhibited in mice with depleted gut microbiota seven days after injury, with these effects lasting for one month [238].

## 8. Targeted Therapies for the Gut–Brain Axis

### 8.1. Fecal Microbiota Transplantation (FMT)

Given the critical role of gut microbiota in neurological diseases, restoring a healthy microbiome by replacing dysbiotic microbial communities may represent an effective therapeutic strategy. FMT involves transferring functional microbiota from the feces of healthy donors into the patient’s gastrointestinal tract to restore gut microbiota and treat both intestinal and extra-intestinal diseases. FMT has shown promising efficacy in gastrointestinal disorders such as inflammatory bowel disease [239].

Preclinical studies suggest FMT’s potential in neurological diseases. For instance, in APPswe/PS1dE9 transgenic mouse models, FMT improves cognitive function by increasing synaptic markers, reducing Aβ accumulation, and mitigating neuroinflammation [240]. In autism spectrum disorder mouse models, FMT from healthy individuals significantly reduces anxiety-like repetitive behaviors and enhances levels of serum factors associated with neurogenesis and synaptic transmission in the CNS [241]. Additionally, FMT plays a critical role in hyperglycemia-induced hemorrhagic transformation (HT), as MCAO rats receiving “HT microbiota” transplants exhibit increased HT risk [242].

Clinical studies on FMT in neurological diseases are still in the exploratory phase. In a study of 54 PD patients, oral FMT significantly improved PD-related autonomic symptoms, gastrointestinal function, and microbiome complexity after three months of intervention [243]. A case report showed that four weeks of oral frozen FMT capsules alleviated depressive symptoms from moderate/severe to mild levels in two patients [244]. Research by Engen et al. demonstrated that FMT intervention significantly increased the abundance of anti-inflammatory butyrate-producing bacteria and SCFA metabolites in multiple sclerosis patients, with sustained elevation of serum BDNF levels and improved walking ability [245].

Currently, FMT application in neurological diseases remains largely at the preclinical stage. Further standardization and optimization of FMT procedures, as well as identification of its active components, are essential for future personalized therapies, providing viable solutions for a range of diseases.

### 8.2. Probiotics and Prebiotics

Probiotic administration aims to introduce specific microbial strains to stimulate healthy microbiome pathways and increase the production of beneficial metabolites [246]. Probiotics restore disrupted microbial communities by competitively excluding specific bacteria, producing antimicrobial peptides, and adhering to the intestinal mucosa. They also strengthen the mucosal barrier by promoting mucin production, preventing the attachment of opportunistic bacteria.

Experimental studies have shown that Morinda officinalis-derived prebiotic FOS effectively restores acetylcholine levels in D-galactose-induced AD rat models by reducing AChE levels [247]. An increased abundance of Bifidobacterium and Lactobacillus upregulates neurotrophic factor expression, preserves postsynaptic density, reduces anxiety-like behaviors, and prevents synaptic damage in colitis mouse models [248]. Combined administration of Pediococcus pentosaceus and prebiotic polygalacturonic acid has shown potential in alleviating gut dysbiosis, reducing GABA levels in MPTP-induced mouse brains, and improving motor dysfunction and neurodegeneration [249]. Engineered GLP-1 probiotic strains mitigate MPTP-induced neurodegeneration by inhibiting glial activation, oxidative stress, and ferroptosis [250]. Clostridium butyricum alleviates cognitive impairment and neuronal damage in SAE mice by reducing microglial activation, decreasing Iba-1 levels, increasing BDNF expression, and improving gut dysbiosis [251].

Clinical studies show that combining probiotics with conventional therapies improves treatment outcomes in PD. Compared to conventional therapy alone, probiotics increase GABA-synthesizing SGB and reduce GABA-degrading SGB [252]. In a randomized, double-blind, controlled trial involving 60 AD patients, 12 weeks of probiotic supplementation (Lactobacillus acidophilus, Lactobacillus casei, Bifidobacterium bifidum, and Lactobacillus fermentum) improved cognitive function and specific metabolic markers [253].

Although the neuroprotective potential of probiotics has been established, determining the optimal dosage to ensure sustained neuroprotection remains challenging. Future studies should investigate the effective therapeutic window and potential long-term consequences of probiotic administration [254].

## 9. Future Directions

A substantial body of research has demonstrated a strong connection between the gut microbiome and various nervous system diseases, including neurodegenerative disorders, neuroinflammatory conditions, and neurological injuries. Interventions targeting the gut microbiota, including probiotics, fecal microbiota transplantation, and antibiotic therapy, have shown potential in modifying disease progression. Thus, the gut microbiome is a promising target for therapeutic intervention in neurological disorders, with changes in gut microbiota potentially serving as biomarkers for these diseases.

Hu et al. conducted a thorough investigation on the effects of incorporating quercetin gel into the diets of mice with RIBI, evaluating its influence on their gut microbiota. This intervention not only modulated the gut microbiome but also led to significant improvements in behavioral outcomes, such as increased spontaneous activity, enhanced short-term memory, and reduced anxiety in treated mice. However, quercetin did not improve cognitive deficits in a mouse model with depleted gut flora, indicating that its protective effects on RIBI are mediated through gut microbiota modulation [255]. Similarly, another study found a decrease in the levels of Mycobacterium phylum, Mycobacteriaceae, and an increase in Heterobacterium spp. levels in a mouse model of RIBI post-WBRT. Enhancing the gut microbiota through antibiotics or probiotics alleviated RIBI in these mice, suggesting a strong association between abnormal gut microbiota composition and WBRT-induced CD. Therefore, strategies aimed at improving gut microbiota composition might benefit individuals with CD exposed to WBRT [256].

Under normal conditions, both the gut and blood–brain barriers are tightly regulated, with immune signals related to tissue microbes being transmitted to the brain [257]. However, the BBB becomes compromised in patients receiving intracranial radiation [258], increasing the likelihood of gut microbiota and their metabolites influencing the nervous system. Although therapies for RIBI are still being explored, targeted regulation of the gut microbiome offers new therapeutic avenues.

Presently, the connection between imbalanced gut microbiota and RIBI has been confirmed in the relevant literature, although most investigations rely heavily on mouse models. Much remains to be explored in clinical research. Recent studies have also emphasized the potential role of gut microbiota and its metabolites in cancer therapy [259,260,261]. The importance of maintaining a balance between the gut microbiome during tumor treatment and managing its associated therapeutic drawbacks should not be disregarded.

To sum up, given our understanding of RIBI mechanisms and advances in gut flora research in other neurological diseases, the role of the gut microbiota in treating RIBI appears significant. However, further in-depth investigation into the molecular mechanisms is necessary to fully realize the promise of these treatments.

## 10. Conclusions

As cancer treatment advances and patient survival rates increase, clinical research is increasingly focused on managing treatment side effects, such as those from radiotherapy. Despite ongoing research, the pathophysiological mechanisms underlying RIBI remain elusive, with chronic vascular damage and neuroinflammation being increasingly posited as plausible explanations.

A recent study by Prof. Tang’s group found that initiating treatment within three months of the initial diagnosis of RIBI can reduce the likelihood of death from any cause by 52% compared to an observation and waiting strategy. This significant finding underscores the need for a more proactive approach to treating RIBI [262].

While the gut–brain axis has not been extensively explored in the context of RIBI, emerging evidence suggests a significant involvement of the gut microbiota in the development of various neurodegenerative diseases. It is conceivable, therefore, that the microbiota could also play a crucial role in RIBI. Targeting the gut microbiota could represent an innovative strategy for treating RIBI.

However, further research is necessary to validate this hypothesis and identify the most effective methods to modulate the gut microbiota for this purpose. Future studies should focus on elucidating the mechanisms through which the gut microbiota influence brain health and determine how these interactions might be harnessed to mitigate the harmful effects of radiation therapy.
